# Supplementary material for: A benchmark for automatic medical consultation system: frameworks, tasks and datasets
Source: Bioinformatics. 2022 Dec 20;39(1):btac817. doi: 10.1093/bioinformatics/btac817 (PMC9848052; doi:10.1093/bioinformatics/btac817)
Supplement: btac817_Supplementary_Data [file btac817_supplementary_data.pdf]

Supplementary Material for A Benchmark for Automatic Medical Consultation System: Frameworks, Tasks and Datasets

In this material, we add some details that can not be presented in the body due to page limitations, including comparisons between DialoAMC and other medical (dialogue) datasets, some examples of designed annotation schema, extension of MRG task, and more experimental details.

1. COMPARISONS WITH PRIOR DATASETS

We have discussed some prior medical dialogue datasets in the relevant work section. To more intuitively show the difference between DialoAMC and other datasets, we present more detailed information about these datasets in Table S1.

MedDialog [1] and KaMed [2] are unlabeled medical dialogue datasets, these datasets are widely available on online medical communities. Dialogue response generation is a common task based on these datasets. MZ [3] and DX [4] are two structured medical dialogue datasets for symptom-based diagnostic policy learning, where each sample is a list of symptoms and their attributes in the dialogue. CMDD [7] and MIE [8] are two dialog datasets for entity state recognition, which annotate medical entities and their statuses (or attributes). MSL [5] and KUAKE-QIC [6] is two medical datasets for slot filling, where the intention of each utterance is labeled, and KUAKE-QIC comes from the browser's search records rather than medical conversations. Dr.Summarize [9] is a dataset for medical report generation, where the report (or summary) of each dialogue are written by medical doctors. MedDG [10] is a large medical dialogue dataset with various annotated medical entities.

Most prior datasets focus on a specific task, so there is a problem of medical label insufficiency. In contrast, DialoAMC contains multi-level annotations, namely token level, utterance level, and dialogue level. They represent the medical concepts in the dialogue from different granularity and perspectives, including named entities (NE), dialog acts (DA), entity attributes (EA), and medical reports (MR). The entity attribute refers specifically to the symptom label in this paper, but it can cover a wider range. These annotations support different tasks covering the modeling of medical conversations, which make DialoAMC a strongly competitive dataset in both annotation granularity and scale.

2. ANNOTATION DETAILS

To give readers a clearer understanding of our annotation schema, we demonstrate examples of medical named entity category and dialogue act category in Table S2 and Table S3 respectively.

3. EXTENSION OF MRG TASK

Intuitively, MRG tasks can benefit from multi-level annotations, including named entities, dialogue acts, and symptom labels. We conduct additional experiments to make use of the information provided by the token level and utterance level to assist in generating medical reports.

Specifically, during training, we endow each utterance with ground truth dialog acts and entities in a markdown-like format (if the entity is a symptom, its label is also included). All utterances whose dialog act category is not *OTHER* are converted and concatenated as input. We demonstrate an example in Figure S1.

In the testing phase, We use the baseline models in the three preceding tasks to predict these labels, i.e., ERNIE-Health for NER and DAC, MC-BERT-MLC for SLI-EXP and MC-BERT-MTL for SLI-IMP. We use ProphetNet as the generation model for MRG task, denoted as ProphetNet-Joint. We also conduct some ablation experiments to verify which information is useful for MRG tasks. We report the results on Table S4.

It can be seen that adding multi-level information is effective. ProphetNet-Joint is superior to ProphetNet in all five metrics. However, the results of ablation experiments find that containing

Table S1. Comparison between DialoAMC and other medical (dialogue) datasets in terms of annotation scale and granularity, where NE, DA, EA and MR are short for Named Entity, Dialog Act, Entity Attribute and Medical Report respectively, and PEDS, Cardio and GE are short for Pediatrics, Cardiology and Gastroenterology.

Dataset	Domain	Scale			Granularity			
		#Diseases	#Dialogues	#Entities	NE	DA	EA	MR
MedDialog [1]	-	172	3.6M	-				
KaMed [2]	-	-	60K	-				
MZ [3]	PEDS	4	710	70			✓	
DX [4]	PEDS	5	527	46			✓	
MSL [5]	-	-	1,652	29	✓	✓		
KUAKE-QIC [6]	-	-	10,880	-		✓		
CMDD [7]	PEDS	4	2,067	161	✓		✓	
MIE [8]	Cardio	6	1,120	71	✓		✓	
Dr.Summarize [9]	-	-	1,690	-				✓
MedDG [10]	GE	12	17,864	160	✓			
**DialoAMC (Ours)**	PEDS	10	4,116	4,692	✓	✓	✓	✓

Table S2. Examples of each medical named entity category.

Entity Category	Abbreviation	Example
Symptom	*SX*	*chest tightness, dysuria, itching, etc.*
Drug Name	*DN*	*medilac-vita, smecta, clindamycin, etc.*
Drug Category	*DC*	*anti-inflammatories, cold medicines, probiotics, etc.*
Examination	*EX*	*blood routine, X-ray, CRP analysis, etc.*
Operation	*OP*	*infusion, nebulization, vaccination, etc.*

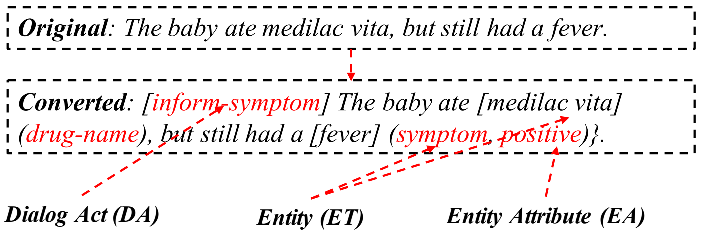

Fig. S1. Multi-level annotations augmented utterance.

Table S3. Examples of each dialog act category, where D and P are short for Doctor and Patient, respectively.

DA Category	Abbreviation	Example
Request Basic Information	*R-BI*	*D: How old is the baby?*
Inform Basic Information	*I-BI*	*P: Ate some lean meat.*
Request Symptom	*R-SX*	*D: Is this kid still vomiting?*
Inform Symptom	*I-SX*	*P: He has a runny nose and a cough.*
Request Etiology	*R-ETIOL*	*P: Does a bacterial infection cause diarrhea?*
Inform Etiology	*I-ETIOL*	*D: May be related to fatigue and cold.*
Request Existing Exam and Treatment	*R-EET*	*D: When was glycerin enema used?*
Inform Existing Exam and Treatment	*I-EET*	*P: Have been taking antiviral drugs.*
Diagnose	*DIAG*	*D: The baby may have an upper respiratory infection.*
Request Drug Recommendation	*R-DR*	*P: There is cough syrup at home. Can the baby drink it?*
Inform Drug Recommendation	*I-DR*	*D: Just drink some probiotics.*
Request Medical Advice	*R-MA*	*P: Is it useful to do pediatric massage?*
Inform Medical Advice	*I-MA*	*D: Yes, a blood test is required to the cause of the infection.*
Request Precautions	*R-PRCTN*	*P: Can i take her to bathe?*
Inform Precautions	*I-PRCTN*	*D: Appropriate to give the child more water to drink.*
Other	*OTR*	*P: Got it, thank you.*

Table S4. Extension of MRG task.

Models	R-1	R-2	R-L	C-F1	RD-Acc
ProphetNet	60.48	45.73	56.41	49.48	61.90
ProphetNet-Joint	**60.81**	**46.16**	**56.82**	49.82	**64.00**
w/o DA	60.40	45.95	56.77	**52.25**	57.50
w/o ET	59.81	45.56	56.21	51.08	62.83
w/o EA	60.31	45.88	56.15	48.62	62.56

both DA and ET have some negative effects on C-F1, since there is some redundancy in these two levels of labels. Besides, we find that the lack of DA will significantly reduce RD-Acc. We guess that the recognition of utterances with DA type as *Diagnosis* does a great help.

4. EXPERIMENTAL DETAILS

To ensure the repeatability of the experiment, we list all the baseline models in Table S5, including the model architecture, and batch size, learning rate and training epochs during training.

For LSTM models listed in the table, we use the same settings. The embedding size of word, character and bi-word is set to 300. For Lattice LSTM and TextRNN, the hidden size is 128, the TextRCNN is 256, and the Seq2seq and PG are 256. The number of hidden layers of all LSTM models is 1, except that TextRNN is 2; For CNN model, the dimension of word vector is set to 300, and other parameters are consistent with the official implementation; For BERT models, we use the same bert-base architecture, i.e., 12 successive transformer layers, each having 12 self-attention heads, and the hidden size is 768. The main difference between them lies in the different initialization parameters, such as *bert-base-chinese*, *ernie-1.0-base-zh* and *ernie-health-chinese*; For Transformer models (vanilla Transformer, T5, ProphetNet and Bio-ProphetNet), we adopt the large version, i.e., the encoder and decoder both contain 12 transformer block layers, the hidden size is 1024, and each contains 16 self-attention heads; For other modules, such as CRF and MLP, their parameters are all randomly initialized.

REFERENCES

1. G. Zeng, W. Yang, Z. Ju, Y. Yang, S. Wang, R. Zhang, M. Zhou, J. Zeng, X. Dong, R. Zhang *et al.*, "Meddialog: Large-scale medical dialogue dataset," in *Proceedings of the 2020 Conference on Empirical Methods in Natural Language Processing (EMNLP)*, (2020).
2. D. Li, Z. Ren, P. Ren, Z. Chen, M. Fan, J. Ma, and M. de Rijke, "Semi-supervised variational reasoning for medical dialogue generation," in *Proceedings of the 44th International ACM SIGIR Conference on Research and Development in Information Retrieval*, (2021), pp. 544–554.
3. Z. Wei, Q. Liu, B. Peng, H. Tou, T. Chen, X.-J. Huang, K.-F. Wong, and X. Dai, "Task-oriented dialogue system for automatic diagnosis," in *Proceedings of the 56th Annual Meeting of the Association for Computational Linguistics (Volume 2: Short Papers)*, (2018), pp. 201–207.
4. L. Xu, Q. Zhou, K. Gong, X. Liang, J. Tang, and L. Lin, "End-to-end knowledge-routed relational dialogue system for automatic diagnosis," in *Proceedings of the AAAI Conference on Artificial Intelligence*, vol. 33 (2019), pp. 7346–7353.
5. X. Shi, H. Hu, W. Che, Z. Sun, T. Liu, and J. Huang, "Understanding medical conversations with scattered keyword attention and weak supervision from responses," in *Proceedings of the AAAI Conference on Artificial Intelligence*, vol. 34 (2020), pp. 8838–8845.
6. N. Zhang, M. Chen, Z. Bi, X. Liang, L. Li, X. Shang, K. Yin, C. Tan, J. Xu, F. Huang *et al.*, "Cblue: A chinese biomedical language understanding evaluation benchmark," arXiv preprint arXiv:2106.08087 (2021).
7. X. Lin, X. He, Q. Chen, H. Tou, Z. Wei, and T. Chen, "Enhancing dialogue symptom diagnosis with global attention and symptom graph," in *Proceedings of the 2019 Conference on Empirical Methods in Natural Language Processing and the 9th International Joint Conference on Natural Language Processing (EMNLP-IJCNLP)*, (2019), pp. 5033–5042.
8. Y. Zhang, Z. Jiang, T. Zhang, S. Liu, J. Cao, K. Liu, S. Liu, and J. Zhao, "Mie: A medical information extractor towards medical dialogues," in *Proceedings of the 58th Annual Meeting of the Association for Computational Linguistics*, (2020), pp. 6460–6469.
9. A. Joshi, N. Katariya, X. Amatriain, and A. Kannan, "Dr. summarize: Global summarization of medical dialogue by exploiting local structures," arXiv preprint arXiv:2009.08666 (2020).
10. W. Liu, J. Tang, J. Qin, L. Xu, Z. Li, and X. Liang, "Meddg: A large-scale medical consultation dataset for building medical dialogue system," arXiv preprint arXiv:2010.07497 (2020).

Table S5. Model architecture and training parameters.

Task	Model	Arch	Batch Size	Learning Rate	Epochs
NER	Lattice LSTM	LSTM+CRF	1	0.015	10
	BERT-CRF	BERT+CRF	64	5e-5	10
	ERNIE	BERT	64	6e-5	10
	FLAT	BERT	32	1e-5	10
	LEBERT	BERT	32	1e-5	10
	MC-BERT	BERT	64	1e-5	10
	ERNIE-Health	BERT	64	6e-5	10
DAC	TextCNN	CNN	128	1e-3	20
	TextRNN	LSTM	128	1e-3	10
	TextRCNN	LSTM	128	1e-3	10
	DPCNN	CNN	128	1e-3	20
	BERT	BERT	128	5e-5	5
	ERNIE	BERT	64	3e-5	5
	MC-BERT	BERT	128	5e-5	5
	ERNIE-Health	BERT	64	3e-5	5
SLI-EXP	BERT-MLC	BERT	128	1e-5	200
	MC-BERT-MLC	BERT	128	1e-5	200
SLI-IMP	BERT-MLC	BERT	128	1e-5	50
	MC-BERT-MLC	BERT	128	1e-5	50
	BERT-MTL	BERT	32	1e-5	20
	MC-BERT-MTL	BERT	32	1e-5	20
MRG	Seq2seq	LSTM	64	1e-3	20
	PG	LSTM	32	1e-3	20
	Transformer	Transformer	64	1e-4	10
	T5	Transformer	64	2e-4	10
	ProphetNet	Transformer	64	1e-4	10
	Bio-ProphetNet	Transformer	64	1e-4	10
DDP	UB-SVM	SVM	-	-	-
	DQN	MLP	100	5e-4	5000
	KR-DQN	MLP	32	1e-2	5000
	REFUEL	MLP	64	1e-4	5000
	GAMP	LSTM+MLP	64	1e-4	1000
	HRL	MLP	100	5e-4	5000
